# A systematic review of transitions between cigarette and smokeless tobacco product use in the United States

**DOI:** 10.1186/s12889-015-1594-8

**Published:** 2015-03-18

**Authors:** Jamie Tam, Hannah R Day, Brian L Rostron, Benjamin J Apelberg

**Affiliations:** Department of Health Management and Policy, University of Michigan, School of Public Health, Ann Arbor, USA; US Food and Drug Administration, Center for Tobacco Products, Silver Spring, USA

**Keywords:** Smokeless tobacco, Smoking, Tobacco, Longitudinal, Transitions

## Abstract

**Background:**

Smokeless tobacco use is becoming an increasingly important public health issue in the US and may influence cigarette smoking behavior. Systematic information on transitions between smokeless tobacco and cigarette use in the US is limited.

**Methods:**

We conducted a systematic review of published literature on transitions between smokeless tobacco and cigarette use in the US. We searched PubMed, Web of Science and EbscoHost databases for all published articles from January 2000 to March 2014 that presented estimates of transitions in US youth and adult study populations over time between at least one of the following tobacco use states: exclusive cigarette smoking, exclusive smokeless tobacco use, dual use of both products, and use of neither product. We excluded non-English language studies, studies published before 2000, clinical trials, controlled cessation programs, and clinical studies or evaluations of smokeless tobacco cessation programs.

**Results:**

The review identified six studies on US populations published since 2000 with longitudinal data on some or all of the transitions that users can undergo between smokeless tobacco and cigarette use. There was considerable heterogeneity across studies in design and tobacco use definitions. Despite these differences, the existing data indicate that switching behaviors from exclusive smoking to exclusive smokeless tobacco use are limited (adults: 0%-1.4%, adolescents: 0.8%-3.8%) but may be more common from exclusive smokeless tobacco use to exclusive smoking (adults: 0.9%-26.6%, adolescents: 16.6%-25.5%). Among adults, exclusive cigarette smoking was generally stable and consistent (79.7% to 87.6%) over follow-up across studies but less stable in adolescents (46.8%-78.7%). Exclusive smokeless tobacco use was less stable than exclusive cigarette smoking over time (adults: 59.4%-76.6%, adolescents: 26.2%-44.8%).

**Conclusion:**

This review provides published estimates of the proportions of adults and adolescents transitioning between tobacco use categories from the most recently available studies on longitudinal transitions between smokeless tobacco and cigarettes in the US. These data can be used to track tobacco use behaviors and evaluate their effect on public health; however, the data for these studies were generally collected more than a decade ago. Additional research including nationally representative longitudinal estimates using consistent definitions and designs, would improve understanding of current tobacco transition behaviors.

## Background

Smokeless tobacco is a commonly used tobacco product in the US. According to the US National Adult Tobacco Survey (NATS), 6.5% of US adult males were current users of chewing tobacco, snuff, or dip and 2.5% were current users of snus in 2009–2010, making smokeless tobacco one of the most commonly used tobacco product among US adults after cigarettes and cigars [1]. Many smokeless tobacco users, particularly those at younger ages, tend to use the product along with other forms of tobacco. Among US high school students, 6.4% of students overall and 11.2% of males were current users of smokeless tobacco, defined as chewing tobacco, snuff, and dip, in 2012 [2], but only 1.6% of US high school students currently used smokeless tobacco as their exclusive tobacco product with the remainder of users using smokeless in combination with one or more other tobacco products [3].

The 2009 Family Smoking Prevention and Tobacco Control Act amended the Federal Food Drug and Cosmetic Act to grant the US Food and Drug Administration (FDA) the authority to regulate cigarettes, smokeless tobacco and roll-your-own tobacco [4]. The Act stipulates that the FDA should, when making certain regulatory decisions concerning tobacco products, consider the impacts of decisions on the population as a whole, including impacts on the likelihood of initiation of tobacco use among non-users and cessation among users [4]. An understanding of tobacco use behavior, including the extent to which individuals transition between products, provides useful information in this context.

In addition to conventional forms of smokeless tobacco such as chewing tobacco and snuff, other smokeless products such as snus and dissolvables have been introduced into the US market in recent years and have attracted attention. Snus is a smokeless tobacco product designed for oral use that was developed in Sweden in the early 19^th^ century [5]. Patterns of snus use in Sweden differ substantially from those in the US [5]. Although market data from Nielsen show that sales of snus in the US doubled between 2009 and 2010 [6], the prevalence of snuff and chewing tobacco use is higher than the prevalence of snus use in the US [1].

Syntheses of information regarding the use and effects of smokeless tobacco products in the US are thus particularly relevant and timely for a variety of audiences. Reviews have been conducted concerning the health effects of smokeless tobacco products including snus for a variety of medical conditions [7-14]. Systematic information on tobacco use behavior involving smokeless tobacco products is much more limited, however. The dynamics of smokeless tobacco use can be complicated, with some users adopting dual or poly-use with other tobacco products, others switching between smokeless and other tobacco products, and still others continuing to use smokeless tobacco exclusively.

This study presents a systematic review of studies of transitions between smokeless tobacco and cigarette use in the US from a longitudinal perspective. Much of the previous literature on the subject has been cross-sectional in nature, focusing on the prevalence of dual cigarette and smokeless tobacco use or the self-reported order of product use, such as studies examining whether smokeless tobacco use increases the likelihood for subsequent smoking. In contrast, this review focuses on longitudinal studies of the transitions to and from smokeless tobacco in order to more fully capture the natural trajectory of smokeless tobacco use in the US population. We identified studies that estimated transitions to and from four product use categories (exclusive smokeless tobacco use, exclusive cigarette smoking, dual cigarette and smokeless use, and use of neither product) between two time points. Because the marketplace for smokeless tobacco products has changed in the US in recent years [6], we restricted our review to studies that have been published since 2000.

## Methods

### Search strategy

We conducted a search using PubMed, Web of Science, and EbscoHost databases for all published articles from January 1^st^, 2000 to March 4^th^, 2014 with the following search terms in the title, abstract, or keywords: 'smokeless tobacco’, ‘snuff’, ‘chewing tobacco’, ‘dip tobacco’, ‘dipping tobacco’, ‘spit tobacco’, or ‘snus’ and either ‘initiation’, ‘cessation’, ‘quitting’, ‘quit’, ‘substitution’, ‘dual use’, ‘dual users’, ‘dual tobacco use’, ‘dual tobacco users’, ‘polytobacco’, ‘gateway’, ‘switch’, ‘switching’, ‘transition’, or ‘harm reduction.’ Records from all databases were combined and duplicates were removed resulting in 1053 total articles. In the first stage of screening, JT and HRD used an internal protocol to screen the titles and abstracts for all records according to the selection criteria outlined in Table 1. To ensure that reported transition behaviors reflected norms and patterns of use in US populations, studies conducted outside the US (such as those in Northern Europe and Southeast Asia) were excluded. In the second stage of screening, they conducted full-text review of each relevant article.

**Table 1:** **Selection criteria for studies**

**I. Inclusion criteria**		**II. Exclusion criteria**	
a.	Original peer-reviewed articles from US studies published between January 1, 2000 and March 4, 2014	a.	Studies from clinical trials, controlled cessation programs, and clinical studies or evaluations of smokeless tobacco cessation interventions without additional follow-up beyond the originally planned timeframe of the program
b.	Articles include estimates of at least one transition between two points in time between a combination of the following tobacco use states: exclusive cigarette smoking, exclusive smokeless tobacco use, dual use of both products, and use of neither product.	b.	Studies that do not have data on smokeless transition behaviors
		c.	Non-human studies
		d.	Non-English language studies
		e.	Commentaries or non-empirical research
		f.	Studies published prior to 2000
		g.	Studies on e-cigarettes as a “smokeless” product

### Data extraction

**Figure 1:**
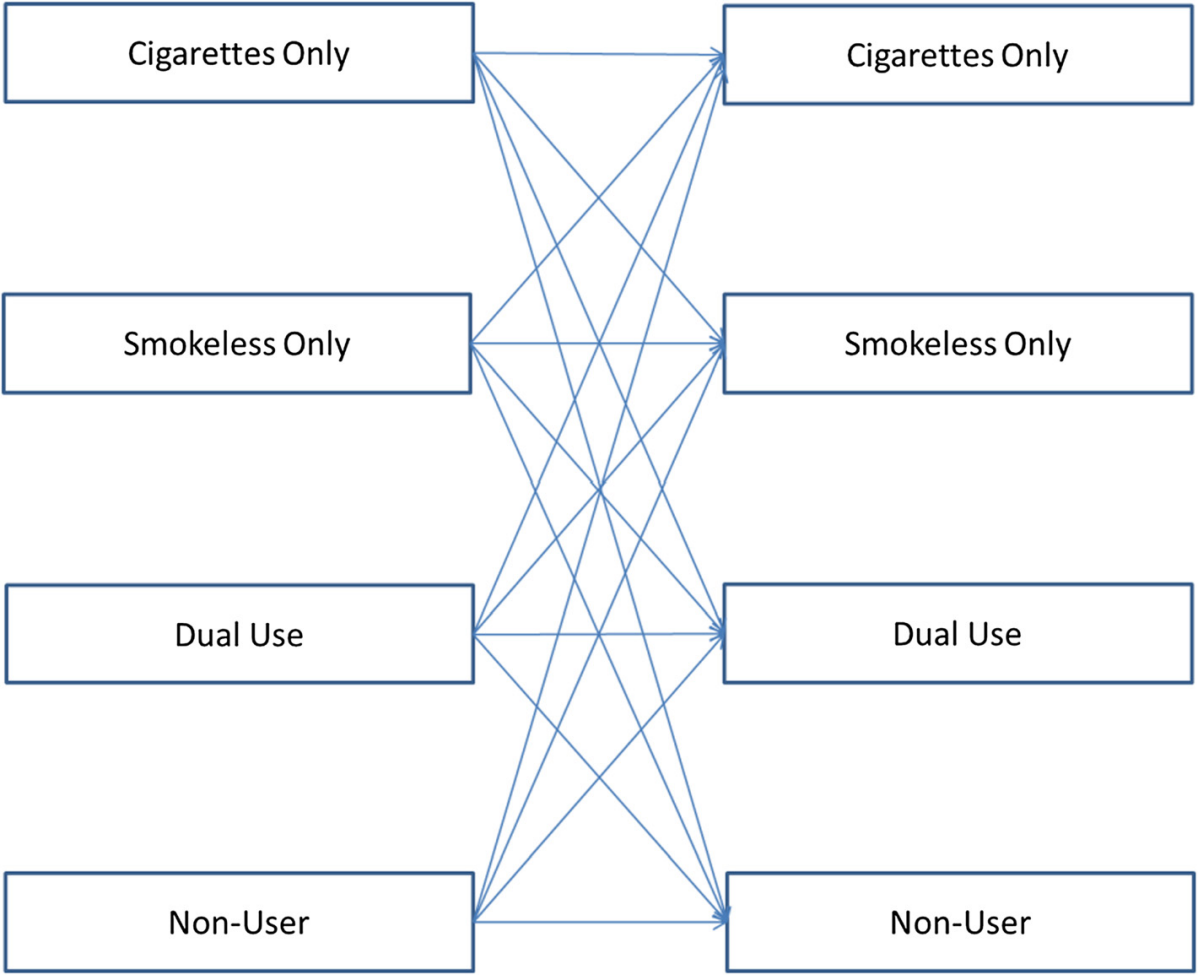
**Conceptual diagram of tobacco product use transitions.**

We extracted information on the proportion of the sample population transitioning from baseline to follow-up between at least one combination of the following tobacco use categories: exclusive smokeless use, exclusive cigarette smoking, dual use of both products, and use of neither product. JT and HRD also independently extracted information on study characteristics including study population, follow-up, definitions for each of the tobacco use categories, and how transitions were calculated. JT and BJA agreed upon data extraction. The conceptual diagram in Figure 1 illustrates the transitions of interest.

## Results

### Study selection

**Figure 2:**
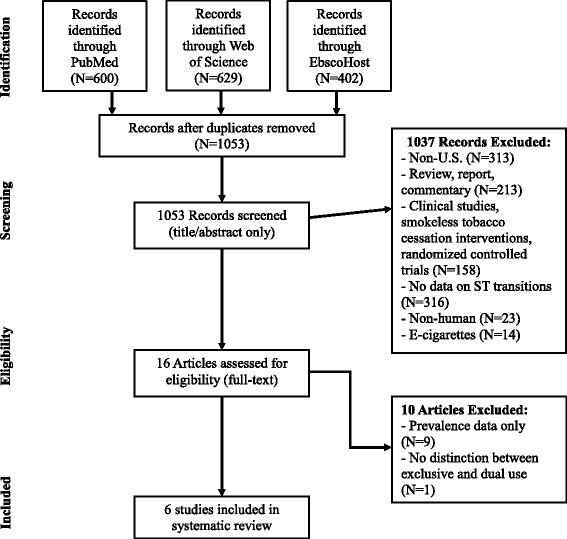
**Study selection flowchart.**

Figure 2 illustrates the different stages of the review process. Searches of the three relevant databases identified 1053 unique articles covering the time period from January 1, 2000 to March 4, 2014. During review of the abstracts, 23 articles were excluded as non-human studies, 158 articles were excluded for evaluating smokeless tobacco cessation interventions without additional follow-up beyond the originally planned timeframe of the intervention or using smokeless tobacco as a smoking cessation aid, 213 articles were excluded as non-empirical studies such as reviews, reports, or commentaries, 313 articles were excluded for describing studies not conducted in the US, 14 articles were excluded for focusing on e-cigarettes (articles which describe e-cigarettes as “smokeless” products), and 316 articles were excluded because they had no data on the tobacco transition states of interest. Of the 16 articles assessed for full text review, nine articles were from cross-sectional studies and only reported prevalence estimates and one article was from a study that did not distinguish between dual use and exclusive use of smokeless tobacco. Six articles met all selection criteria and were included in the review.

### Study characteristics as sources of heterogeneity

Our results indicate that nearly every study used different study populations, tobacco use definitions, and follow-up times in producing estimates of tobacco product use transitions. These differences can affect estimates and comparisons of results across studies. For this reason, we did not conduct meta-analysis to integrate reported transition estimates quantitatively. Tables 2 and 3 summarize the key characteristics of the six selected studies. Three of the studies were conducted among adults, and three studies were conducted among adolescents.

**Table 2:** **Study characteristics - adults**

**Study**	**Study design**	**Population**	**Follow-Up and Loss to Follow-Up**	**Definition: use neither product**	**Definition: exclusive smokeless tobacco (ST) user**	**Definition: exclusive smoker**	**Definition: dual use**	**How transitions calculated**
Zhu et al. (2009)	• Tobacco Use Supplement to Current Population Survey 2002–2003 (TUS CPS)	• n = 15,056	• Follow-up one year later in 2003	Non-user = does not currently use either product, subgroups include never smokers and former smokers	ST user = currently uses chewing tobacco or snuff every day or some days	Cigarette smoker = has smoked ≥ 100 cigarettes in lifetime and currently smokes cigarettes every day or some days	Dual user = ST user + cigarette smoker	Percentages are weighted by census-derived survey weights, and stratified by gender, but not adjusted for other factors.
		• Males and females	• Only includes data for adults with baseline and follow-up information (excludes 1.9% of the sample with conflicting smoking information)					
	• Nationally representative cross sectional survey with longitudinal component in some cycles	• ages 18+						
		• Nationally representative						
	• Household interviews at baseline in 2002 with follow-up in 2003 for some participants							
Wetter et al. (2002)	• Secondary trial data from Working Well cancer prevention trial – University of Texas MD Anderson Cancer Center and worksites	• n = 1244 tobacco users	• Follow-up four years later	Non-user = has not smoked in past seven days and does not currently use smokeless tobacco	ST user = currently uses chewing tobacco, dip, or snuff	Cigarette smoker = has smoked ≥ 100 cigarettes in lifetime and has smoked in past seven days	Dual user = ST user + cigarette smoker	percentages are unadjusted
		• Males	• Only includes employees who remained at their baseline worksite four years later (62% of baseline sample)					adjusted odds ratios, for other analyses (not presented here)
	• Baseline in 1990	• Average age = 37.5 years						
		• Reside in southeastern U.S.	• 52% of baseline dual users had follow up data, compared to 60% of smokers and 66% of ST users.					
Haddock et al. (2001)	• Secondary data from Wilford Hall/University of Memphis and Minnesota Smoking Cessation Program	• n = 7865	• Mail-in follow-up one year after military basic training	Non-user = Never smoker + never ST user or never smoker + former ST user	ST user = uses smokeless tobacco at least once per day	Cigarette smoker at follow-up = has smoked at least a puff in last seven days	n/a	percentages are unadjusted
		• Males						
	• Baseline questionnaire in August 1995	• Average age 19.8 years	• Follow-up takes place after an imposed tobacco ban	Never Smoker = has never smoked regularly (at least one cigarette per day)				adjusted odds ratios for other analyses (not presented here)
	• Treatment and control groups during a 6-week imposed tobacco ban in August 1996	• U.S. Air Force young adult male recruits who reported being never smokers at baseline	• 96% of smokers and 66% of nonsmokers had follow-up data from parent study					
			• Follow-up excludes those who dropped out of basic training, completed training but dropped out of the survey, were deceased, or on assignment)

**Table 3:** **Study characteristics - adolescents**

**Study**	**Study design**	**Population**	**Follow-up and loss to follow-up**	**Definition: use neither product**	**Definition: exclusive ST user**	**Definition: exclusive smoker**	**Definition: dual use**	**How transitions calculated**
Tomar et al. (2003)	• The Teenage Attitudes and Practices Survey (TAPS-I and II), nationally representative cohort study, in 1988–1989 and 1993	• n = 3,996	• Four-year follow-up between 1989 and 1993	Non-user = Not ST user + Not current smoker	ST user = self-identification as regular user of chewing tobacco or snuff	Current smoker = smoked ≥ 100 cigarettes in lifetime and smoked at least 1 day in 30 days preceding interview	Dual user = ST user + current smoker	percentages are weighted by survey weights
		• Young males						
	• Telephone interviews and self-administered questionnaires by mail (TAPS-I) or in-person contact (TAPS-II)	• Ages 11-19	• 87.1% of baseline sample completed follow-up					adjusted odds ratios for other analyses (not presented here)
		• Nationally representative						
Severson et al. (2007)	• Cohort study conducted between 1994 and 1999	• n = 2263	• Two-year follow-up in 9th or 11th grade	Non-user = Non-smoker + Not ST user	ST user = any smokeless tobacco use in the past 30 days	Current smoker = WSI score is ≥ 1	Dual user = ST user + cigarette smoker (only included at follow-up)	percentages are unadjusted
		• Young males						
	• Baseline survey completed once in 7th or 9th grade	• 7th and 9th-graders at baseline		Non-smoker = Has never smoked and Weekly Smoking Index (WSI) score is 0. WSI score averages answers to four questions about current smoking during past month				adjusted odds ratios for other analyses (not presented here)
	• Evaluation of a randomized community intervention to prevent adolescent use	• Small rural communities in Oregon						
O’Hegarty et al. (2012)	• National Longitudinal Study of Adolescent Health (Add Health), a nationally representative school-based sample with Wave I conducted during the 1994–1995 school year	• n = 3284	• Wave II conducted in 1996, approximately 1–2 years after Wave I (1994–1995)		ST user = reported using smokeless tobacco during the past 30 days	Current smoker = smoked on at least 1 of the past 30 days	Dual user = smoked on at least 1 of the past 30 days and used reported using smokeless during the past 30 days	
		• Male and female						
		• Grades 7–11 when interviewed in Wave I						
		• Nationally representative sample						

In the studies of adults, one study provided transition data from a nationally representative study population. Zhu et al. [15] used data from 15,056 men and women participating in the Tobacco Use Supplement to the Current Population Survey (TUS-CPS) in 2002 and 2003. Other studies of adults used data from more specific study populations. Wetter et al. [16] presented data from 1,244 adult male tobacco users in the Working Well Trial in 1990 with follow-up four years later. The Working Well Trial was a large multi-center cancer prevention study designed to examine the effectiveness of comprehensive worksite health promotion interventions related to diet and tobacco use. Data in the Wetter et al. study come from the one study center in which participants initially received information on smokeless tobacco cessation in addition to information on diet and smoking, although follow-up extended beyond this intervention period. Haddock et al. [17] presented data from 7,865 male never smoking US Air Force recruits in the Wilford Hall/University of Memphis and Minnesota Smoking Cessation Program in 1995 with follow-up one year later. In the initial intervention program, participants were randomly assigned to a smoking cessation program or control group during a six-week period in which tobacco use was prohibited for both groups. The average age of participants in the Haddock et al. study was 19.8 years.

In studies of adolescents, two of the three studies used nationally representative study populations, and study sizes ranged from 2,263 to 3,996 participants. The two nationally representative studies were O’Hegarty et al. [18], which used data from waves I (1994–1995) and II (1996) of the National Longitudinal Study of Adolescent Health (Add Health) and Tomar et al. [19], which used data from the Teenage Attitudes and Practices Survey (TAPS-I and II) from 1989 with follow-up four years later. Severson et al. [20] used data from the Project Sixteen study conducted from 1994–1999 in rural Oregon with follow-up two years after baseline. Project Sixteen randomized school-based tobacco prevention programs in Oregon communities to receive community-wide programs that incorporate mass media, family communications, and other activities [21].

The results presented in this review are for the observed proportions of individuals who transition from one product use category to the same or different category at follow-up. There is some inconsistency between the data presented in the figures and tables in Severson et al. [20], and we specifically used transition proportions from the figures. Wetter et al. [16], Haddock et al. [17], O’Hegarty et al. [18], Tomar et al. [19], and Severson et al. [20] also presented adjusted odds ratios for transition between product use categories, but those odds ratios are not the focus of the current review.

### Follow-up conditions differ by study

The studies included in this review included only individuals with both baseline and follow-up information, and differences in loss to follow-up could have affected the transition probabilities. For example, follow-up in the Wetter et al. [16] study was limited to employed men who still worked at their original site of employment, thus allowing for the possibility of healthy worker bias or other types of bias related to employment. The Haddock et al. [17] study similarly had follow-up for study participants who had remained in the military for one year.

### Tobacco use definitions varied across studies

Tobacco use definitions varied across studies, as shown in Tables 2 and 3. In adults, Zhu et al. [15] and Wetter et al. [16] defined cigarette smokers as individuals who had smoked at least 100 cigarettes in their lifetimes and either currently smoked every day or some days in Zhu et al. [15] or had smoked in the past seven days in Wetter et al. [16]. Haddock et al. [17] defined current smokers as those who had smoked, even a puff, over the past seven days. For smokeless tobacco use in adults, Zhu et al. defined smokeless tobacco use as use of chewing tobacco or snuff every day or some days; Wetter et al. defined smokeless tobacco use as current use of chewing tobacco, dip or snuff; and Haddock et al. defined current smokeless tobacco use as use at least once per day. Dual use in Zhu et al. and Wetter et al. was defined as the combination of the smoking and smokeless tobacco use definitions for each study, and Haddock et al. did not include dual users. Tobacco product use transitions were calculated as the proportion of adults who reported one category of tobacco product use at baseline and then the same or different category at follow-up.

In studies of adolescents, O’Hegarty et al. [18] defined smokers as having smoked on at least one of the past 30 days, Tomar et al. [19] defined current smokers as individuals who had smoked at least 100 cigarettes in their lifetimes and had smoked on at least one of the past 30 days, and Severson et al. [20] classified individuals as current smokers if they scored greater than one on a weekly smoking index. O’Hegarty et al. [18] and Severson et al. [20] defined smokeless tobacco use based on use in the past 30 days and Tomar et al. [19] defined smokeless tobacco use based on self-report of regular use. Adolescent dual users were defined in each of the three studies from the combination of the definitions used in each study for cigarette smokers and smokeless tobacco users. Tobacco product use transitions were again calculated as the proportion of study participants who reported one category of tobacco product use at baseline and then the same or different category at follow-up.

### Transitions between cigarette smoking and smokeless tobacco use states

Table 4 summarizes reported adult transition probabilities between each of the following tobacco use states: exclusive smokeless tobacco use, exclusive cigarette smoking, dual product use, and use of neither product. Table 5 presents reported transition probabilities for adolescents.

**Table 4:** **Percent of adults transitioning between tobacco product use categories, by study**

		**Follow-up status**			
		**Neither**	**Exclusive smokeless tobacco user**	**Exclusive smoker**	**Dual user**
**Baseline**	**Neither**	*1 year follow-up*	*1 year follow-up*	*1 year follow-up*	*1 year follow-up*
		• 73.9%* male former smokers quit ≤1 yr [15]	• 1.7% males former smoker quit ≤1 yr (0.5 to 5.8) [15]	• 24.4% males former smoker quit ≤1 yr (17.1 to 33.6) [15]	• 0% males former smoker quit ≤1 yr [15]
		• 97%* males former smoker quit >1 yr [15]	• 0.3% males former smoker quit >1 yr (0.1 to 0.7) [15]	• 2.6% males former smoker quit >1 yr (1.9 to 3.6) [15]	• 0.1% males former smoker quit >1 yr (0.0 to 0.5) [15]
		• 96.7%* males never smoker [15]	• 0.7% males never smoker (0.5 to 1.1) [15]	• 2.5% males never smoker (1.7 to 3.8) [15]	• 0.1% males never smoker (0 to 0.3) [15]
		• 68.3%* females former smoker quit ≤1 yr [15]	• 0% females former smoker quit ≤1 yr [15]	• 31.7% females former smoker quit ≤1 yr (24.9 to 39.3) [15]	• 0% females former smoker quit ≤1 yr [15]
		• 97.1%* females former smoker quit >1 yr [15]	• 0.3% females former smoker quit >1 yr (0.1 to 0.6) [15]	• 2.9% females former smoker quit >1 yr (2.1 to 4.0) [15]	• 0% females never smoker [15]
				• 1.7% females never smoker (1.2 to 2.2) [15]	
		• 98.3%* females never smoker [15]	• 0% females never smoker [15]	• 12.9% of male never ST users [17]	
				• 26.3% of male former ST users [17]	
	**Exclusive smokeless tobacco user**	*1 year follow-up*	*1 year follow-up*	*1 year follow-up*	*1 year follow-up*
		• 35.0% males (27.0 to 43.8) [15]	• 59.4% males (50.6 to 67.7) [15]	• 3.9% males (1.4 to 10.6) [15]	• 1.8% males (0.6 to 5.5) [15]
		• 44.1% females (22.2 to 68.6) [15]	• 52.7% females (27.4 to 76.7) [15]	• 3.2% females (0.2 to 31.5) [15]	• 0% females [15]
				• 26.6% males [17]	
		*4 year follow-up*	*4 year follow-up*	*4 year follow-up*	*4 year follow-up*
		• 20.1% males [16]	• 76.6% of males [16]	• 0.9% males [16]	• 2.5% of males [16]
	**Exclusive smoker**	*1 year follow-up*	*1 year follow-up*	*1 year follow-up*	*1 year follow-up*
		• 11.3% males (8.7 to 14.2) [15]	• 0.3% males (0.1 to 0.8) [15]	• 86.2% males (83.1 to 88.9) [15]	• 2.2% males (1.4 to 3.5) [15]
		• 12.3% females (10.2 to 14.7) [15]	• 0% females [15]	• 87.6% female (85.2 to 89.7) [15]	• 0.1% female (0.0 to 0.2) [15]
		*4 year follow-up*	*4 year follow-up*	*4 year follow-up*	*4 year follow-up*
		• 15.7% males [16]	• 1.4% males [16]	• 79.7% males [16]	• 3.2% males [16]
	**Dual user**	*1 year follow-up*	*1 year follow-up*	*1 year follow-up*	*1 year follow-up*
		• 13.1% males (4.9 to 30.7) [15]	• 4.9% males (0.9 to 23.1) [15]	• 37.0% males (23.2 to 53.4) [15]	• 45.0% males (29.7 to 61.3) [15]
		• 0% females [15]	• 0% females [15]	• 71.6% females (14.0 to 97.5) [15]	• 28.4% females (2.5 to 86.0) [15]
		*4 year follow-up*	*4 year follow-up*	*4 year follow-up*	*4 year follow-up*
		• 11.3% of males [16]	• 17.4% of males [16]	• 27.0% males [16]	• 44.3% of males [16]

*Calculated from other estimates provided in paper.

[15] Zhu et al., nationally representative Tobacco Use Supplement to the Current Population Survey.

[16] Wetter et al., secondary data from the Working Well Trial.

[17] Haddock et al., secondary data from the Wilford Hall/University of Memphis and Minnesota Smoking Cessation Program (US Air Force).

**Table 5:** **Percent of adolescents transitioning between tobacco product use categories, by study**

		**Follow up status**			
		**Neither**	**Exclusive smokeless tobacco user**	**Exclusive smoker**	**Dual user**
**Baseline**	**Neither**	*2 year follow-up*	*2 year follow-up*	*2 year follow-up*	*2 year follow-up*
		• 71.5% males [20]	• 4.6% males [20]	15.7% males [20]	• 8.3% males [20]
		*4 year follow-up*	*4 year follow-up*	*4 year follow-up*	*4 year follow-up*
		• 82.2% males [19]	• 3.1% males [19]	• 13.5% males [19]	• 1.1% males [19]
	**Exclusive smokeless tobacco user**	*2 year follow-up*	*2 year follow-up*	*2 year follow-up*	*2 year follow-up*
		• 16.6% males [20]	• 26.2% males [20]	• 16.6% males [20]	• 40.7% males [20]
		*4 year follow-up*	*4 year follow-up*	*4 year follow-up*	*4 year follow-up*
		• 15.2% males [19]	• 44.8% males [19]	• 25.5% males [19]	• 14.3% males [19]
	**Exclusive smoker**	*1-2 year follow-up*	*1-2 year follow-up*	*1-2 year follow-up*	*1-2 year follow-up*
		• 20.0% males and females combined [18]	• 1.6% males and females combined [18]	• 73.4% males and females combined [18]	• 5% males and females combined [18]
		*2 year follow-up*	*2 year follow-up*	*2 year follow-up*	*2 year follow-up*
		• 25.6% males [20]	• 3.8% males [20]	• 46.8% males [20]	• 23.8% males [20]
		*4 year follow-up*	*4 year follow-up*	*4 year follow-up*	*4 year follow-up*
		• 16.9% males [19]	• 0.8% males [19]	• 78.7% males [19]	• 3.6% of males [19]
	**Dual user**	*1-2 year follow-up*	*1-2 year follow-up*	*1-2 year follow-up*	*1-2 year follow-up*
		• 17.9% males and females combined [18]	• 8.5%* males and females combined [18]	• 36.6% males and females combined [18]	• 37% males and females combined [18]
		*4 year follow-up*	*4 year follow-up*	*4 year follow-up*	*4 year follow-up*
		• 14.1% males [19]	• 34.2% males [19]	• 31.2% males [19]	• 20.4% males [19]

*Calculated from other estimates provided in the paper.

[18] O’Hegarty et al., nationally representative National Longitudinal Study of Adolescent Health (Add Health).

[19] Tomar et al., nationally representative Teenage Attitudes and Practices Survey.

[20] Severson et al., secondary data from Project Sixteen (rural Oregon) (transition estimates calculated from Figures 1 and 2).

### Transitions among non-users at baseline

Use of neither tobacco product was the most stable tobacco use state. The studies estimating transitions for baseline non-tobacco users among adults were Zhu et al. [15] and Haddock et al. [17]. Haddock et al. included male military recruits who did not use cigarettes or smokeless tobacco at baseline. After a period of military basic training in which tobacco use was not permitted, study participants were then allowed to use tobacco products. After one year, 12.9% of never smokeless tobacco users and 26.3% of former users had begun smoking cigarettes. Zhu et al. used data from TUS-CPS, and separated non-users into three groups, never smokers, those who had quit smoking in the past year, and those who had quit smoking more than a year prior. At one year follow-up, nearly a quarter of male former smokers and slightly less than a third of female former smokers who had quit smoking in the past year began smoking cigarettes again. Few never smokers or former smokers who had quit more than a year ago transitioned to cigarette smoking at one year follow-up. Few males or females who were not using tobacco in 2002 transitioned to smokeless in 2003. The proportion of male and female non-users at baseline transitioning to dual use was zero or near zero at follow-up.

Among adolescents, most non-tobacco users at baseline also remained non-users at follow-up. Two studies presented estimates of transitions among baseline non-users. Tomar et al. [19] found that 13.5% of baseline non-users had transitioned to smoking after four years, whereas Severson et al. reported a figure of 15.7% after two years. Low rates of smokeless tobacco initiation were seen among adolescent non-users in both studies. Transition estimates for dual use varied, however, between the two studies. Tomar et al. found that 1.1% of non-users had transitioned to dual use after four years, whereas Severson et al. found that 8.3% of non-users had transitioned to dual use after two years.

### Transitions among exclusive smokeless tobacco users at baseline

The percentage of exclusive smokeless tobacco adult users transitioning to cigarette smoking varied by study and may depend on the definition for current smoking. Of the three studies among adults with transition estimates for smokeless tobacco users at baseline, both Zhu et al. [15] and Wetter et al. [16] found that few adults (<5%) switched from exclusive smokeless tobacco use to exclusive cigarette use or dual use after one and four years, respectively. However, Haddock et al. [17] found that a much larger share of male exclusive smokeless tobacco users (26.6%) had become cigarette smokers at one year of follow-up. Both Zhu et al. and Wetter et al. used more stringent definitions for current smoking (“has smoked at least 100 cigarettes in lifetime and currently smokes” or “has smoked in the last week”), but Haddock et al. defined current smoking as having smoked at least a puff in the last seven days. Zhu et al. and Wetter et al. also found that many baseline exclusive smokeless tobacco users did not report any tobacco use at follow-up, with estimates ranging from 20.1% to 44.1% depending on the study group.

Adolescent exclusive smokeless tobacco use was a relatively unstable tobacco use state with less than half of adolescent male exclusive smokeless tobacco users remaining exclusive smokeless tobacco users at follow-up in studies by Tomar et al. [18] and Severson et al. [20]. Both studies found high rates of switching to cigarette use. Tomar et al. reported that 25.5% of baseline smokeless tobacco users had switched to cigarettes after four years, and Severson et al. reported a figure of 16.6% after two years. In Tomar et al., 14.3% of baseline smokeless tobacco users had become dual users compared with 40.7% in Severson et al. Estimates of transition to use of neither product were 15.2% for Tomar et al. and 16.6% for Severson et al.

### Transitions among exclusive cigarette smokers at baseline

Exclusive cigarette smoking among adults was a stable tobacco use state; most adult exclusive cigarette smokers at baseline remained exclusive cigarette smokers at follow-up in Zhu et al. [15] and Wetter et al. [16]. Few exclusive adult smokers transitioned to exclusive smokeless tobacco use or dual use in these studies. The proportion of exclusive cigarette smokers using neither product at follow-up was 11.3% for males and 12.3% for females after a year in Zhu et al. and 15.7% after four years in Wetter et al.

Among adolescents, exclusive smoking may be less stable compared to adults, as results varied more widely. Tomar et al. found that 78.7% of baseline exclusive smokers remained smokers after four years, O’Hegarty et al. found 73.4% after 1–1.5 years, and Severson et al. found 46.8% after two years [18-20]. Few exclusive smokers switched to smokeless tobacco use only in the studies. In the two nationally representative studies, few (≤5%) exclusive cigarette smokers became dual users compared with 23.8% of exclusive smokers in Severson et al. The two nationally representative studies also found lower proportions of baseline cigarette smokers had transitioned to use of neither product (16.9% and 20.0%) compared with Severson et al. (25.6%).

### Transitions among dual ST and cigarette users at baseline

Compared to other tobacco use categories, dual use of both cigarettes and smokeless tobacco was the least stable tobacco use state. Slightly less than half of adult male dual users at baseline remained dual users after one or four years of follow-up in Zhu et al. and Wetter et al. [15,16]. In Zhu et al., 37% of male and 71.6% of female dual users had transitioned to just cigarette use. In Wetter et al., 27% of male dual users had transitioned to just cigarettes. Estimates for male dual users transitioning to smokeless tobacco use only were 4.9% in Zhu et al. and 17.4% in Wetter et al. Estimates for the transition to use of neither product for males were 13.1% in Zhu et al. and 11.3% in Wetter et al.

The majority of adolescent dual users also transitioned to other tobacco use states at follow-up. The two nationally representative studies examined transitions among adolescent dual users, with less than half of dual users remaining dual users at follow-up. O’Hegarty et al. found that 36.6% of dual users transitioned to cigarettes only, compared with 31.2% in Tomar et al. [18,19]. O’Hegarty et al. also found that 8.5% of dual users had transitioned to smokeless tobacco use only, and 17.9% were using neither product at follow-up, whereas Tomar et al. found that 34.2% were using smokeless tobacco only and 14.1% were using neither product.

## Discussion

This systematic review has identified six studies published in the US since 2000 with estimates of transitions that users can make between smokeless tobacco use, cigarette smoking, dual product use, and use of neither product across two time points. To our knowledge, this is the first study to systematically review and report estimates of these important tobacco product use transitions. The review presents transition estimates for adults and adolescents, and for males and females within these age categories.

In general, the estimates presented here suggest that smokeless tobacco use may be less consistent than cigarette smoking, at least based on the definitions and time frames used in the various studies. Most non-tobacco users and exclusive smokers do not transition to other behaviors. For example, among adults, the transitions probabilities for exclusive cigarette smokers were for the most part stable and consistent across categories, as were transition probabilities among never and former smokers who had quit more than a year ago. Consistent with what is known about smoking initiation, estimates from this review show that adults who do not smoke cigarettes (non-tobacco users or exclusive smokeless tobacco users) are unlikely to start, but this is less true for adolescents. Few non-smokers (non-tobacco users or exclusive smokeless tobacco users) report taking up smoking across studies, with the exception of the military study, which reported higher smoking initiation rates across all groups. These results may be related to factors specific to the military recruit population such as young age or high rates of tobacco use in the military.

Many people who report smokeless tobacco use (exclusive smokeless tobacco or dual use) at baseline report not having used smokeless tobacco within a certain period at follow-up. This was particularly noticeable in Zhu et al. [15], where 38.8% of exclusive smokeless tobacco users at baseline reported not using the product at one-year follow-up, whereas far fewer dual users report using smokeless tobacco only or not using either product at follow-up. It is not clear, however, if these results indicate high rates of actual cessation among smokeless tobacco users, or if they reflect that many smokeless tobacco users use the product only occasionally and thus may not report themselves as smokeless tobacco users at certain points in time. Among adolescents, the rural population studied by Severson et al. [20] showed evidence of higher smokeless initiation compared with the nationally representative study populations in Tomar et al. [18]. In both the Tomar et al. [18] and Severson et al. [20] studies, fewer than half of exclusive smokeless tobacco users remained exclusive smokeless tobacco users at follow-up, suggesting that smokeless tobacco use among some adolescents may be a transitory state, although the studies differ on whether these users are more likely to transition to exclusive smoking or dual use. Current tobacco use in adolescent studies is also often assessed by any use in the past 30 days, so classification issues, as well as use behavior, may contribute to instability in use pattern estimates among youth. In the two nationally representative studies, most exclusive smokers remained in this category, although an appreciable number had quit smoking altogether.

Reported transition estimates should be evaluated in the context of study-specific limitations. For example, estimates from some studies may not be generalizable to the US population as a whole. Three of the studies used nationally representative study populations, specifically the Teenage Attitudes and Practices Survey [19], the National Longitudinal Study of Adolescent Health [18], and the Tobacco Use Supplement to the Current Population Survey [15]. The three remaining studies used smaller and non-representative study populations in settings such as the military, as in Haddock et al. [17], or in a particular region, such as Wetter et al.’s study in the southeastern US [16] or Severson et al.’s study in rural Oregon [20].

Tobacco use definitions and follow-up time also varied across studies. Definitions of smokeless tobacco use, for example, included some day or daily use [15], daily use [17], current use [16], self-identification as a regular smokeless tobacco user [19], or reported use of smokeless tobacco within the past 30 days [18,20]. Definitions of current smoking exhibited similar variability across studies.

Follow-up time generally varied from one to four years across studies, and differential loss to follow-up could affect transition estimates. Longer follow-up may reflect more enduring tobacco use states, although it would not capture multiple changes in tobacco behavior within that time period and could also suffer from disproportionate loss to follow-up. Shorter follow-up times could capture more proximate tobacco use state changes but may not reflect stable patterns of use.

There is also the potential for bias in estimates from some of the studies due to the presence of a tobacco use intervention or ban. The Wetter et al. [16], Severson et al. [20], and Haddock et al. [17] studies each utilize data from parent studies that included some type of tobacco intervention. As such, the presence of a cancer prevention program and smokeless tobacco intervention in the Wetter et al. study, smoking cessation interventions in Severson et al. and Haddock et al., and an imposed tobacco ban in Haddock et al. may influence tobacco use behavior and transitions.

Additional limitations of the studies include the absence of confidence intervals for transition estimates and the absence of estimates for females, with the exception of Zhu et al. [15]. Hegarty et al. [18] reported estimates for male and female adolescents combined together.

Even with these limitations however, the results presented in this review can be used by researchers for a variety of purposes. For example, researchers have begun to develop simulations to model changes in tobacco use in populations, including the use of multiple products and allowing for switching between exclusive, dual-, and non-use of products [22,23]. Results from this review can inform the development of these models and also provide key input parameter values for product use. Results from the review can also be used by government agencies and regulatory bodies to better track tobacco use behavior and evaluate its effect on public health.

Smokeless tobacco use is an important public health issue in the US, and information about transitions to and from these products is needed to understand and evaluate their public health impact. Longitudinal studies can best capture these transitions, and nationally representative data provide the most accurate estimates of these transitions at the population level. The use of standardized product use definitions and consistent follow-up time would also enhance estimates and aid in the comparison of results across studies.

Our results highlight the need for continued research in this field. We chose to focus our analysis on the most recently published studies of smokeless tobacco use transitions but also conducted a search of studies published between January 1, 1990 and December 31, 1999 as sensitivity analysis. This search found no articles from this period that met our selection criteria.. Our search identified six relevant studies, but the selected studies sometimes analyzed data collected in the 1990’s or early 2000’s. Given that tobacco use behaviors can change over time, the transition patterns reported here may not reflect newer developments in the smokeless tobacco market, including the rising consumption of snus and moist snuff [6]. The fact that our results represent the only recent longitudinal estimates available is a strong indication of the need for additional studies on tobacco use transitions in the US.

New sources of tobacco use data will become available in the future. In the US, the National Institutes of Health (NIH) and the Food and Drug Administration (FDA) are currently conducting the Population Assessment of Tobacco and Health (PATH) study, a longitudinal cohort study of tobacco use in the US population, which may provide additional data on tobacco use transitions. Baseline data collection for PATH began in 2013, and follow-up data will be collected on an annual basis. In the interim, the longitudinal transition estimates presented here provide useful insight into the way individuals use these particular products in an increasingly diverse tobacco use environment. Our review compiles the most recent transition estimates published to date and can be used for comparison with these forthcoming data.

## Conclusion

This systematic review has presented and analyzed the transition estimates for cigarette and smokeless tobacco use for US youth and adults that are currently available in the research literature. These estimates provide important information about tobacco product use and transitions that can be used in a variety of contexts including tobacco use modeling. Specific estimates of transition probabilities were sometimes substantially different across studies, and the reasons for these differences may include differences in study population characteristics, use of different tobacco use definitions, and varying lengths of follow-up.
